# Opposing Signaling of ROCK1 and ROCK2 Determines the Switching of Substrate Specificity and the Mode of Migration of Glioblastoma Cells

**DOI:** 10.1007/s12035-013-8568-6

**Published:** 2013-10-30

**Authors:** Sonja Mertsch, Solon Thanos

**Affiliations:** Institute for Experimental Ophthalmology, School of Medicine, Westfalian-Wilhelms-University Münster, Albert-Schweitzer-Campus 1, D15, 48149 Münster, Germany

**Keywords:** Glioblastoma multiforme, Cell lines, Migration, Stripe assay, Cell signaling

## Abstract

Despite current advances in therapy, the prognosis of patients with glioblastoma has not improved sufficiently in recent decades. This is due mainly to the highly invasive capacity of glioma cells. Little is known about the mechanisms underlying this particular characteristic. While the Rho-kinase (ROCK)-dependent signaling pathways involved in glioma migration have yet to be determined, they show promise as one of the candidates in targeted glioblastoma therapy. There are two ROCK isoforms: ROCK1, which is upregulated in glioblastoma tissue compared to normal brain tissue, and ROCK2, which is also expressed in normal brain tissue. Blockage of both of these ROCK isoforms with pharmacologic inhibitors regulates the migration process. We examined the activities of ROCK1 and ROCK2 using knockdown cell lines and the newly developed stripe assay. Selective knockdown of either ROCK1 or ROCK2 exerted antidromic effects on glioma migration: while ROCK1 deletion altered the substrate-dependent migration, deletion of ROCK2 did not. Furthermore, ROCK1 knockdown reduced cell proliferation, whereas ROCK2 knockdown enhanced it. Along the signaling pathways, key regulators of the ROCK pathway are differentially affected by ROCK1 and ROCK2. These data suggest that the balanced activation of ROCKs is responsible for the substrate-specific migration and the proliferation of glioblastoma cells.

## Introduction

Glioblastoma multiforme (World Health Organization grade IV), which is the most common brain tumor in humans, has a median survival time of only 12–14 months. One reason for this poor prognosis is the ability of single tumor cells to invade diffusely into the neighboring brain parenchyma. After tumor resection followed by adjuvant radiation and chemotherapy, 90 % of patients are subject to recurrences within months, usually in the tissue adjacent to the resection area [1]. Therefore, any efficient therapeutic approach must reach cells that have invaded far beyond the radiologically and intraoperatively visible borders because cells migrate along the white matter tracts and basement membranes of blood vessels [2–6].

Key players in the process of glioma migration seem to be the Ras homolog gene family (Rho)-associated protein kinases (ROCKs) [7–9]. The two ROCK isoforms, ROCK1 and ROCK2, act downstream of the small GTPase Rho member A (RhoA). The ROCK1 and ROCK2 peptides display some similarities in the kinase activity domain at the N-terminus, the coiled-coil domain, and a pleckstrin homology (PH) domain at the C-terminus. GTP-bound RhoA activates the Rho kinases by displacement of the PH domain, thus enabling different substrates to bind to the kinase domain [10, 11]. LIM kinase (LIMK) is activated by phosphorylation through ROCKs. This activation leads to a phosphorylation of cofilin, which in turn inhibits actin depolymerization, leading to a consequent increase in actin polymerization. Furthermore, ROCKs also phosphorylate the myosin-binding subunit of myosin phosphatase, leading to the inactivation of the phosphatase activity [12]. ROCKs also mediate—at least in part—a translocation of Rho signaling to the nucleus, where Rho regulates the functions of various transcription factors including the four and a half LIM domains protein (FHL2) and estrogen receptor (ER) [13, 14]. While integrins and matrix metalloproteases are typically involved in the mesenchymal type of migration, ROCKs are involved in amoeboid movement and migration [15].

Recently, we have established a co-culture migration assay that allows cells to migrate along myelinated axons, with a view to examine the molecular mechanisms underlying tumor cell migration along white matter tracts using pharmacological ROCK inhibition [16]. Furthermore, we established a modified stripe assay to determine the substrate specificity changes of glioma cells under ROCK inhibition by using the unselective inhibitor Y27632 [17].

In the present study, we investigated whether the shRNA-induced inhibition of either ROCK1 or ROCK2 alone influences glioma migration and proliferation and elucidated the differences between these two ROCKs in glioblastoma cell migration, substrate preferences, and cell proliferation. We were able to show that ROCK1 alone is capable of inducing the migratory effects and ROCK2 displays controversy effects of ROCK1.

## Materials and Methods

### Cell Lines

Human glioblastoma cell lines U87MG, U343MG, 86HG39, U373MG, D54MG, H4, T98G, and A172 (all cell lines are kind gifts of V. Senner, Institute of Neuropathology, Muenster, Germany) were cultured in Dulbecco’s modified Eagle’s minimal essential medium (DMEM) with 10 % fetal calf serum, 100 U/ml penicillin, and 100 μg/ml streptomycin at 37 °C in 5 % CO_2_ (all cell culture reagents were purchased from PAA, Linz, Austria).

### Stable Transfection

Four different shRNA oligonucleotides for ROCK1 inhibition [sure silencing shRNA plasmid ROCK1 hygromycin (KH01966H)], including one negative control vector and four different shRNA oligonucleotides for ROCK2 knockdown [sure silencing shRNA plasmid ROCK2 hygromycin (KH09606H)] were purchased from SA Biosciences (Hilden, Germany). Cells were seeded at a density of 0.8 × 10^5^ in 24-well plates with 500 μl of culture medium the day before shRNA transfection. Cells were transfected by adding Attractene Transfection Reagent (Qiagen) according to the manufacturer’s recommendations and maintained in culture medium for 24 h before selection with 600 μg/ml hygromycin. After colony formation, at least 60 independent clones were chosen for each vector sequence. The extent of RNA knockdown under these conditions was determined with quantitative real-time polymerase chain reaction (qRT-PCR) analyses, Western blotting, and immunostaining.

### Immunoblotting

Lysis of cells and tissue, sodium dodecyl sulfate polyacrylamide gel electrophoresis, and immunoblotting were conducted as described [17]. Rabbit anti-ROCK1 antibody (Sigma-Aldrich, 1:1,000 dilution), rabbit anti-ROCK2 antibody (Sigma-Aldrich, 1:1,000 dilution), anti-rabbit phospho-LIMK1/LIMK2 (Cell Signaling, 1:1,000 dilution), anti-rabbit phosphoCdc42/rac (Cell Signaling, 1:1,000 dilution), anti-mouse RhoA (Abcam, 1:300 dilution), anti-rabbit phosphor-Akt1 (Abcam, 1:1,000), anti-mouse Akt1 (Abcam, 1:300 dilution), anti-mouse cyclin D1 (Abcam, 1:1,000 dilution), anti-goat β1-integrin (Santa Cruz Biotechnology, 1:200 dilution), anti-mouse β-catenin (BD Biosciences, 1:2,000 dilution), and anti-rabbit phosphoERK1/2 (Cell Signaling, 1:500 dilution) were used. Peroxidase-conjugated goat anti-rabbit (Cell Signaling) or goat anti-mouse secondary antibody (Cell Signaling), at a dilution of 1:5,000 for 1 h at room temperature, was used. To verify equal protein loading on each lane, the blots were stripped and reprobed for rabbit anti-calnexin (Sigma-Aldrich, 1:20,000 dilution), mouse anti-β-actin (Sigma-Aldrich, dilution 1:20,000), or rabbit anti-glyceraldehyde-3-phosphate dehydrogenase (GAPDH; Sigma-Aldrich, 1:200,000 dilution). For analyzing the results, lanes were quantified using densitometry (AlphaEraseFC Software), and loading controls (calnexin, GAPDH, or β-actin) were used for standardization.

### Immunofluorescence

After permeabilizing the cells with 0.1 % Triton X-100 (Sigma) in phosphate-buffered saline (PBS), they were blocked with 0.5 % bovine serum albumin in PBS. The rabbit ROCK1 and ROCK2 antibodies were applied to separate groups of cells at a dilution of 1:100 and incubated at 4 °C overnight. A tetramethylrhodamine/isothiocyanate-conjugated anti-mouse antibody (T1689, Sigma, 1:200 dilution) was used as the secondary antibody. Actin filaments were visualized by incubating the cells for 30 min with fluorescein isothiocyanate (FITC)–phalloidin. The cell nuclei were counterstained with 4′,6-diamidino-2-phenylindole (DAPI) and then mounted in anti-fading medium Mowiol (Merck, Darmstadt, Germany). Fluorescence was documented using an Axiophot microscope (Zeiss) with AxioVision Software (Zeiss).

### qRT-PCR Analyses

Total RNA was isolated from sub-confluent cultured cells using an RNeasy Plus Mini kit (Qiagen). Total RNA (1 μg) was transcribed into cDNA with the High Capacity cDNA Reverse Transcription Kit (Applied Biosystems, Foster City, CA, USA) in a reaction volume of 20 μl. After cDNA synthesis, 1 μl from the reaction volume was used for qRT-PCR measurements with the following SYBR Green primers:

- AAAAATGGACAACCTGCTGC (ROCK1, forward)
- GGCAGGAAAATCCAAATCAT (ROCK1, reverse)
- CGCTGATCCGAGACCCT (ROCK2, forward)
- TTGTTTTTCCTCAAAGCAGGA (ROCK2, reverse)

Relative RNA levels were calculated and compared between shRNA- and control-transfected cells. The data were normalized relative to those for GAPDH using the following primers:

- TGCACCACCAACTGCTTAGC (GAPDH, forward)
- GGCATGGACTGTGGTCATGAG (GAPDH, reverse)

The relative expressions were calculated using the 2^−ΔΔ*C*T^ method. All measurements were conducted in duplicate and the experiments were repeated at least three times.

### Wound-Healing Assay

Cell migration was analyzed using a wound-healing assay. Briefly, cells were seeded at a density of 0.8 × 10^5^ per well in a 24-well plate. After 24 h, cell monolayers were scratched using the back side of a standard 100-μl pipette tip. After being washed three times with PBS, photomicrographs were taken of the scratches, including the flanking front lines of cells (at ×40 magnification), and then incubated under standard conditions. Migration into the scratched area was documented at 24 and 48 h after wounding. Scratch closure by migrating cells was compared between ROCK1/ROCK2 knockdown cells and control-transfected cells. Wound closing was evaluated relative to the total area of wounding by counting the migrating cells using a light microscope (Zeiss) and TScratch Software (CSE Lab, Zurich, Switzerland). Experiments were performed independently three times, with four to eight scratches being evaluated for each experimental condition.

### Monolayer Migration and Proliferation Assay

Permanox LabTek eight-well chamber slides (Nunc, Langenselbold, Germany) were coated with poly-l-lysine and Matrigel solution (BD Biosciences) 24 h before use. The chambers were filled with a 200-μl volume of pre-warmed DMEM, after which sterile glass sedimentation cylinders were placed in each chamber. Cells in DMEM were seeded at a density of 2 × 10^3^ into the lumen of the cylinders in a volume of 2 μl. The cylinders were removed after the cells had been allowed to adhere to the substrate (16–24 h after seeding). The resulting colonies were photographed immediately after removal of the cylinders and again at intervals of 24 and 48 h thereafter. Cell migration was evaluated by measuring the increase in the area of the colonies using ZEN software (Zeiss). The change in the area of each colony at each time point was standardized against the colony area measured from the photograph taken immediately after removal of the cylinders. Cellular proliferation of ROCK1 and ROCK2 knockdown cells was assessed with the 3-(4,5-dimethylthiazol-2-yl)-2,5-diphenyltetrazolium bromide (MTT) assay [18].

### Stripe Assay

The principle of the stripe assay is that the cells can choose between the different surfaces during migration, so that any differences in affinity, motility, and cell proliferation can be observed [17]. The cells were seeded onto the stripped membranes at a concentration of 0.8 × 10^5^ cells/ml and allowed to migrate for 48 h. They were then fixed in 4 % paraformaldehyde and transferred to glass coverslips. The stripes were visualized by staining the substrates with FluoSpheres before they were applied to the membranes; the cells were stained with DAPI after fixation. Cell quantification was achieved by first photographing the membranes (at least 15–20 photographs per membrane) and then counting the cells on the different stripes using ImageJ software. The chosen stripe components were homogenized rat retina free of myelin and myelin-containing perinatal rat brains. Biomatrix (BM), which is commercially available, was chosen as the extracellular matrix (Serva, Mannheim, Germany).

### Statistics

The results are presented as the mean ± SEM percentage values. Statistical significance was analyzed using paired Student’s *t* test; the level of statistical significance was set at *p* < 0.05.

## Results

### Expression Analyses and Stable Knockdown of the ROCKs

**Fig. 1:**
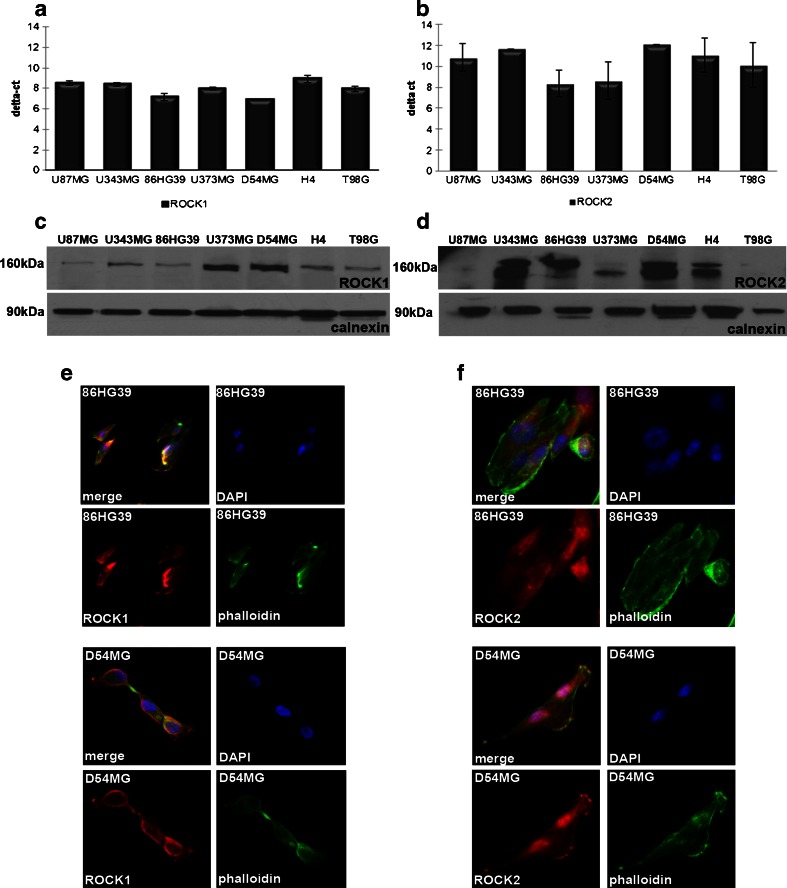
ROCK1 and ROCK2 protein and mRNA expressions in different human glioblastoma cell lines. Quantitative real-time PCR analyses of ROCK1 (**a**) and ROCK2 (**b**) mRNA in different glioblastoma cell lines reveal the highest amount of ROCK1 mRNA in the cell lines 86HG39 and D54MG and the highest amount of ROCK2 mRNA in 86HG39 and U373MG. Western blot analyses of ROCK1 (**c**) and ROCK2 (**d**) protein expressions in commonly used glioblastoma cell lines display an increased ROCK1 protein expression in the cell lines U373MG and D54MG and an increased ROCK2 expression in the cell lines 86HG39, D54MG, and U343MG. Immunofluorescence staining of 86HG39 and D54MG glioma cell lines using antibodies raised against ROCK1 (**e**) and ROCK2 (**f**) and FITC–phalloidin (*green*; to stain the cytoskeleton) shows cytoskeleton- and membrane-associated location of ROCK1 and ROCK2 in both cell lines used

Seven glioma cell lines were first evaluated to screen for the best cell line for stable knockdown of both ROCKs. At the mRNA level, the 86HG39 and D54MG cell lines displayed the strongest expression of ROCK1, while the strongest expressions of ROCK2 were found in the U373MG and 86HG39 cell lines (Fig. 1a, b). The expression levels of ROCK1 and ROCK2 proteins differ from the mRNA results; here, we found the highest expression for ROCK1 in the cell lines D54MG and U373MG and for ROCK2 in D54MG, 86HG39, and U353MG (Fig. 1c, d). Because of the expression levels and the genetic aspects of the cell lines [19, 20], we decided to use D54MG and 86HG39 human glioma cell lines for further investigations. To reveal the cellular location of ROCK1 and ROCK2 in both cell lines, we performed fluorescence immunohistology staining (Fig. 1e, f). Both proteins show a cytoplasmic and membrane-associated location in human glioblastoma cell lines.

**Fig. 2:**
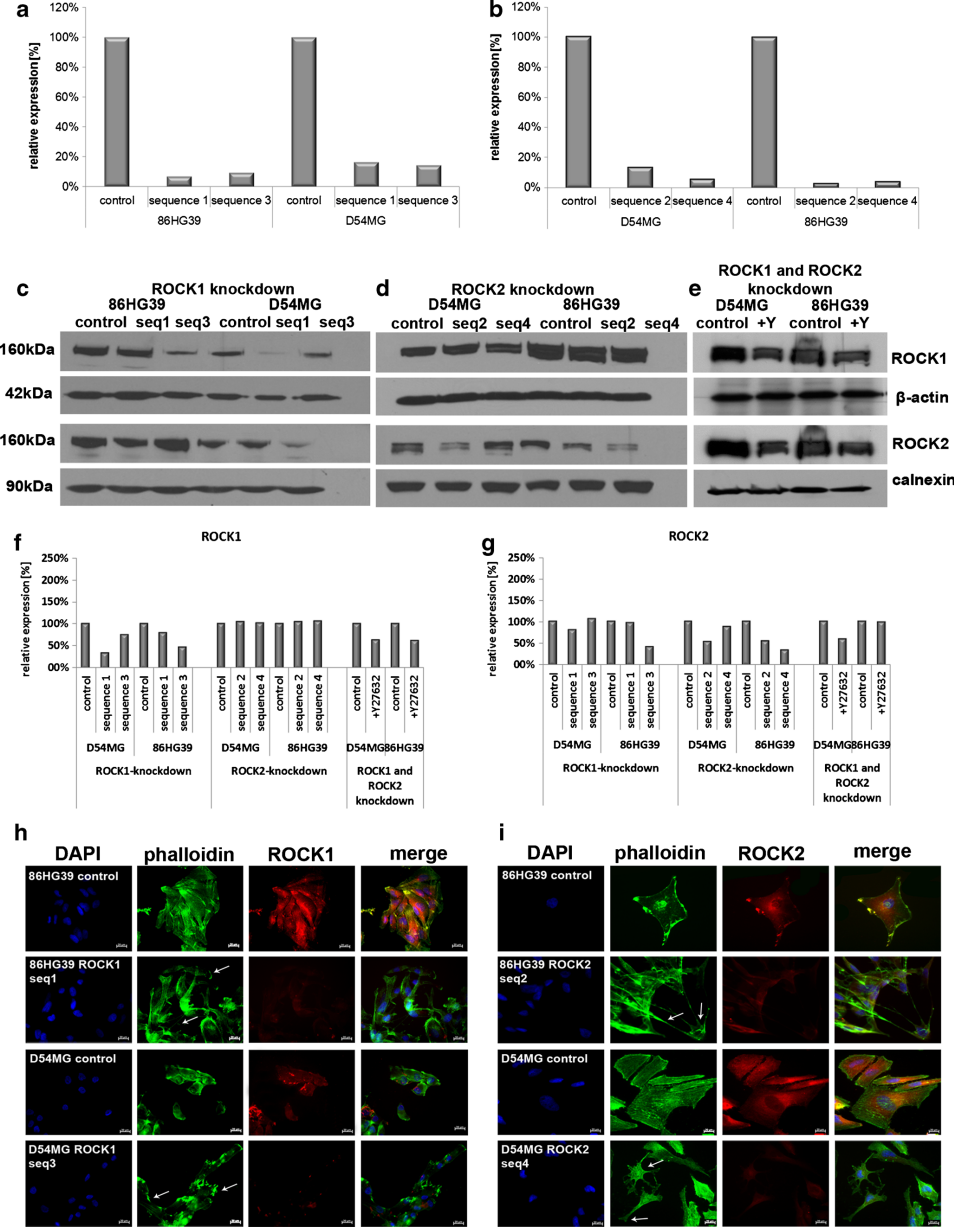
Verification of ROCK1 and ROCK2 knockdown in the human glioblastoma cell lines 86HG39 and D54MG at the mRNA and protein levels. Knockdown of ROCK1 using two different shRNA sequences (seq1 and seq3) and a vector control (set as 100 %) in D54MG and 86HG39 cell lines at the mRNA (**a**) level shows a reduction of ROCK1 mRNA expression level in D54MG cells to 16.6 and 14.4 % and in 86HG39 cells to 7.0 and 9.4 %. On the protein level (**c**), densitometric measurement (**f**) reveals also a reduction in ROCK1 protein expression to 34.0 % (seq1), 74.4 % (seq3) for D54MG, and 79.7 % (seq1) and 47.0 % (seq3) for 86HG39. ROCK2 expression in ROCK1 knockdown clones is also affected as D54MG seq1 reveals a 79.5 % expression of ROCK2 and 86HG39 seq3 of 39.8 %. Quantification of ROCK2 knockdown with two different shRNA sequences (seq2 and seq4) and a control (set as 100 %) at the mRNA level (**b**) reveals a reduced ROCK2 expression levels to 13.4 and 5.7 % for D54MG cells and 2.7 and 3.9 % for 86HG39 cells. On the protein level (**d**, **g**), we found reductions of ROCK2 protein expression to 51.7 % (seq2) and 87.8 % (seq4) for D54MG and 53.9 % (seq2) and 33.2 % (seq4) for 86HG39. ROCK1 protein expression was not influenced by ROCK2 knockdown. Using the inhibitor Y27632 (**e**), we could verify a knockdown of both ROCK1 and ROCK2 on the protein level in both cell lines. Immunofluorescence staining of the 86HG39 and D54MG cell lines with ROCK1 (**h**) and ROCK2 (**i**) knockdown shows changes in cell morphology (indicated by *white arrows*) in ROCK knockdown cells relative to control cells with normal ROCK expression

To avoid off-target effects, we used two different shRNA vector sequences (referred to as seq1 and seq3 for ROCK1 and seq2 and seq4 for ROCK2) and a vector control to induce the knockdown, and at least 60 different clones were screened for each vector construct and each cell line. The reduction of both ROCK1 and ROCK2 expressions in the selected clones was verified using qRT-PCR analyses and Western blotting. ROCK1 mRNA expression in the D54MG cell line was reduced to 16.6 % for sequence 1 clone 4 (D54MG seq1) and to 14.4 % for sequence 3 clone 13 (D54MG seq3). The knockdown of ROCK1 was more efficient in the 86HG39 cell line, with an expression level of 7.0 % for sequence 1 clone 12 (86HG39 seq1) and of 9.4 % for sequence 3 clone 10 (86HG39 seq3; Fig. 2a). ROCK2 mRNA expression in the D54MG cell line was reduced to 13.4 % for sequence 2 clone 2 (D54MG seq2) and to 5.7 % for sequence 4 clone 39 (D54MG seq4); that in the 86HG39 cell line was 2.7 % for sequence 2 clone 52 (86HG39 seq2) and 3.9 % for sequence 4 clone 3 (86HG39 seq4; Fig. 2b). A distinct reduction in the level of protein expression of ROCK1 (Fig. 2c) and ROCK2 (Fig. 2d) was also found in all four clones. D54MG seq1 has a ROCK1 protein level of 34.0 % and seq3 of 74.4 %; in the cell line 86HG39, we found ROCK1 protein levels of 79.7 % (seq1) and 47.0 % (seq3; Fig. 2f). ROCK2 protein level was also affected by ROCK1 knockdown (Fig. 2g). Here, we found a reduced ROCK2 expression in D54MG seq1 (79.5 %) and in 86HG39 seq3 (39.8 %). The knockdown of ROCK2 leads to ROCK2 protein levels of 51.7 and 87.8 % for D54MG seq2 and seq4 and of 53.9 and 33.2 % for 86HG39 seq2 and seq4. Analysis of the ROCK1 protein expression in ROCK2 knockdown clones exhibits no changes. The inhibitor Y27632 affects both kinases ROCK1 and ROCK2. ROCK1 protein expression in the cell line D54MG was reduced to 63.3 % and in the cell line 86HG39 to 61.5 %. ROCK2 protein level shows reduction to 58.1 % for D54MG and only a slight effect on 86HG39 to 98.2 % (Fig. 2e–g).

### ROCK1 and ROCK2 Influence Cell Proliferation and Change Cell Morphology

**Fig. 3:**
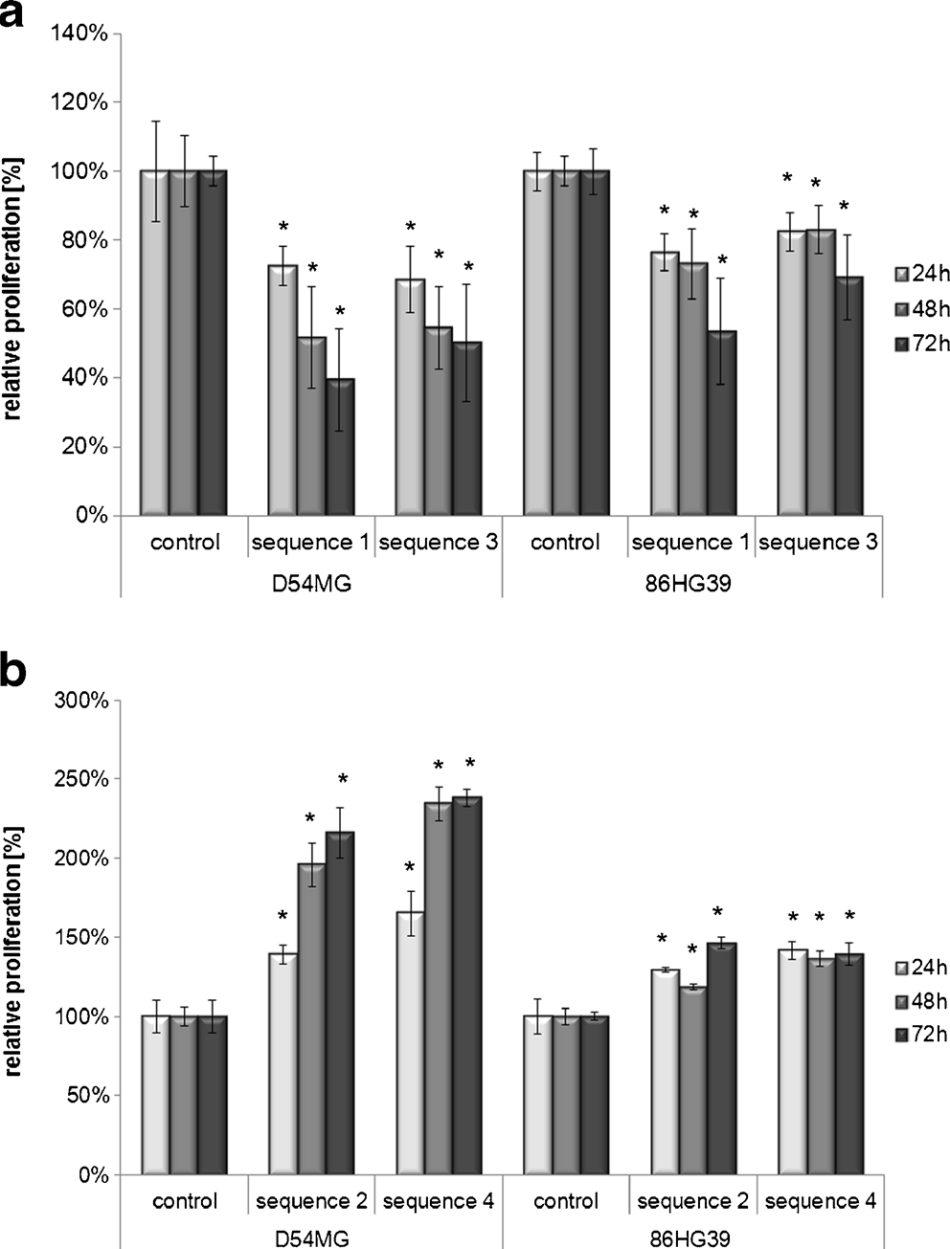
Proliferation analyses of cells with reduced ROCK1 and ROCK2 expressions compared to control cells. The knockdown of ROCK1 led to a significant decrease in cell proliferation in both cell lines (D54MG and 86HG39) and in both cell clones used per cell line (*sequence1* and *sequence3*) relative to the control (set as 100 %) for up to 72 h (**a**), whereas the inhibition of ROCK2 expression in the cell lines led to an increase in proliferative activity (**b**), as assessed with the MTT assay. *Asterisks* represent the representative *p* values (*p < 0.05; **p < 0.001, *n* = 3, mean ± SEM)

Next, we determined whether the effects of ROCK1/ROCK2 knockdown on cell migration are based on changes in cell proliferation. A significant decrease in cellular growth was observed in ROCK1-deficient cells relative to the vector controls (set as 100 %). For all of the used ROCK1 knockdown clones, pronounced changes in proliferation were observed at 72 h (D54MG: sequence 1 = 39.4 ± 14.9 %, sequence 3 = 50.2 ± 16.9 %; 86HG39: sequence 1 = 53.7 ± 15.4 %, sequence 3 = 69.2 ± 12.4 %; Fig. 3a). Conversely, knockdown of ROCK2 enhanced cell proliferation. The maximum proliferation rate was found at 72 h for both cell lines (D54MG: sequence 2 = 216.1 ± 16.0 %, sequence 4 = 238.0 ± 5.4 %; 86HG39: sequence 2 = 130.3 ± 8.0 %, sequence 4 = 147.7 ± 8.3 %; Fig. 3b).

Immunofluorescence staining for ROCK1/ROCK2 and FITC–phalloidin were used to determine whether knockdown of ROCK1 and ROCK2 alters the cellular phenotype. All of the ROCK1 knockdown clones displayed changes in cell morphology and developed a mesenchymal-like phenotype. Inhibition of ROCK1 and ROCK2 led to several cytoskeletal and morphologic changes including inhibition of stress fibers, enhancement of the number and length of cell processes, and an increase in the degree of membrane ruffling (Fig. 2h, i). The knockdown cells displayed a stellar appearance with an increase of the number and length of actin-positive membrane ruffles. These data show that there is no distinct difference in the change in cell phenotype between ROCK1 and ROCK2 knockdown cells.

### Differential Effects on Cell Migration of ROCK1 and ROCK2

**Fig. 4:**
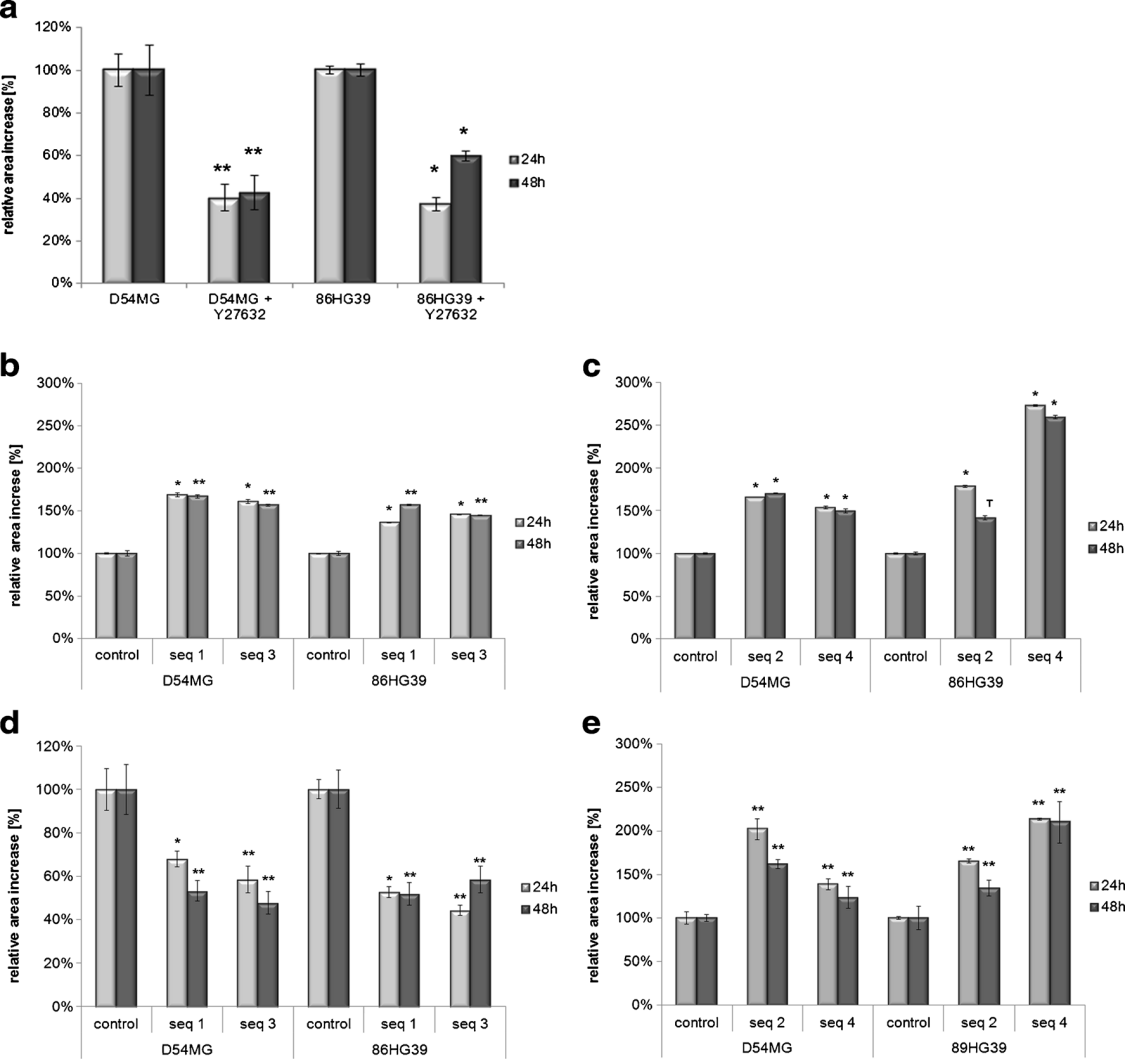
Different migration assays using the D54MG and 86HG39 glioblastoma cell lines with ROCK inhibitor Y27632 and ROCK1/ROCK2 knockdown. In a coated radial monolayer migration assay, the cells displayed a reduction in cell migration when treated with the ROCK inhibitor Y27632 (**a**). Cells with a stable ROCK1 knockdown exhibited enhanced cell migration using a wound-healing assay on an uncoated surface (**b**), but a significant decrease in cell migration using a radial monolayer assay on a laminin-coated surface (**d**). Glioma cell lines with a stable ROCK2 knockdown exhibited an increase in migration on both the uncoated surface (**c**) and the laminin-coated surface (**e**) (*p < 0.05; **p < 0.001, *n* = 3, mean ± SEM)

Functional analysis of the effects of ROCK1 and ROCK2 knockdown on cell migration was conducted with uncoated wound-healing migration assays as well as radial monolayer assays coated with laminin, respectively. Simultaneous inhibition of both ROCK1 and ROCK2 was performed using a monolayer migration assay on a laminin-coated surface with D54MG and 86HG39 cells using the ROCK inhibitor Y27632. A significant reduction in cell migration was found. In the D54MG cell line, the addition of 100 μM Y27632 resulted in migration rates of 46.8 ± 6.4 and 51.3 ± 8.1 % at 24 and 48 h, respectively. In the 86HG39 cell line, simultaneous inhibition of both ROCK1 and ROCK2 reduced the cell migration rates to 38.7 ± 3.5 and 68.2 ± 2.3 % at 24 and 48 h, respectively (Fig. 4a). ROCK1 and ROCK2 knockdown cells were separately subjected to repeated migration assays in order to establish whether the reduction in migration observed when both ROCK1 and ROCK2 are inhibited is based on the reduction of both or whether reduction of either kinase is sufficient to account for the effect.

When ROCK1 protein synthesis was inhibited in the D54MG cell line, the cells migrated faster on an uncoated surface, at rates of 168.9 ± 2.5 % (seq1) and 160.8 ± 2.4 % (seq3) at 24 h and of 167.0 ± 1.9 % (seq1) and 156.8 ± 1.1 % (seq3) at 48 h relative to the migration rate of the control cells (set as 100 %; ±1.0 % at 24 h, ±3.1 % at 48 h). Similar results were observed with the 86HG39 cell line, for which the wound closing rates were 136.2 ± 0.6 % (seq1) and 145.7 ± 0.4 % (seq3) at 24 h and 156.7 ± 0.9 % (seq1) and 144.6 ± 0.6 % (seq3) at 48 h (Fig. 4b). Thus, knockdown of ROCK1 leads to a highly significant increase in cell migration on an uncoated surface. In contrast, ROCK1 knockdown cells displayed a significant decrease in cell migration on the radial monolayer coated with laminin. With the D54MG cell line, there were reductions in the migration rate of 68.0 ± 3.7 % (24 h) and 53.2 ± 4.8 % (48 h) for sequence 1 and of 58.5 ± 6.3 % (24 h) and 47.7 ± 5.2 % (48 h) for seq3 (relative to the control, set as 100 % for all time points). The 86HG39 cell line exhibited a comparable inhibition of cell migration on the laminin-coated surface under ROCK1 knockdown, whereby the migration rates were reduced (relative to control, set as 100 %) to 52.7 ± 2.6 % (24 h) and 51.9 ± 5.3 % (48 h) for seq1 and to 44.3 ± 2.3 % (24 h) and 58.5 ± 6.2 % for seq3 (Fig. 4d). Together, these findings show that ROCK1 knockdown leads to a substrate-dependent migration effect, with enhanced migration on an uncoated surface and reduced migration on a laminin-coated surface (Fig. 4b, d).

In contrast to ROCK1 knockdown, ROCK2 knockdown increased the migration rate in both assays. Migration on the uncoated surface was significantly increased (relative to the control of 100 ± 2.1 %) for both cell lines and for all tested clones, at 141.0 ± 16.8 % for 86HG39 seq4 at 48 h and 273.0 ± 16.8 % for 86HG39 seq2 at 24 h (Fig. 4c). On the radial monolayer migration assay with a laminin-coated surface, the migration rate relative to the control was 123.6 ± 11.8 % for D54MG seq4 at 48 h and 213.6 ± 11.8 % for 86HG39 seq4 at 24 h. These data show that the reduction in ROCK2 expression in both glioblastoma cell lines led to a substrate-independent increase in migration (Fig. 4e). The cell migration effects of the two ROCKs thus differ in that those of ROCK1 are substrate-dependent whereas those of ROCK2 are independent of the substrate.

### Knockdown of ROCK1, But Not ROCK2, Leads to a Significant Change in the Substrate Specificity of Tumor Cells

The stripe assay, which allows cells to be confronted simultaneously with two different substrates [21], was used to further examine the effects of the ROCKs on cell migration. In a previous study, we showed that glioma cells have a distinct preference for the extracellular matrix compared to all other substrates [17]. To reflect the components of an intact 3D brain environment, membrane fractions from unmyelinated rat retina were used to represent gray matter, along with purified myelin and BM, and all three substrates were tested against each other using ROCK1 and ROCK2 knockdown cell lines and the control cell lines. Furthermore, the ROCK inhibitor Y27632 was applied to the ROCK1 knockdown cells and the controls to elucidate whether Y27632 has an additive effect.

**Fig. 5:**
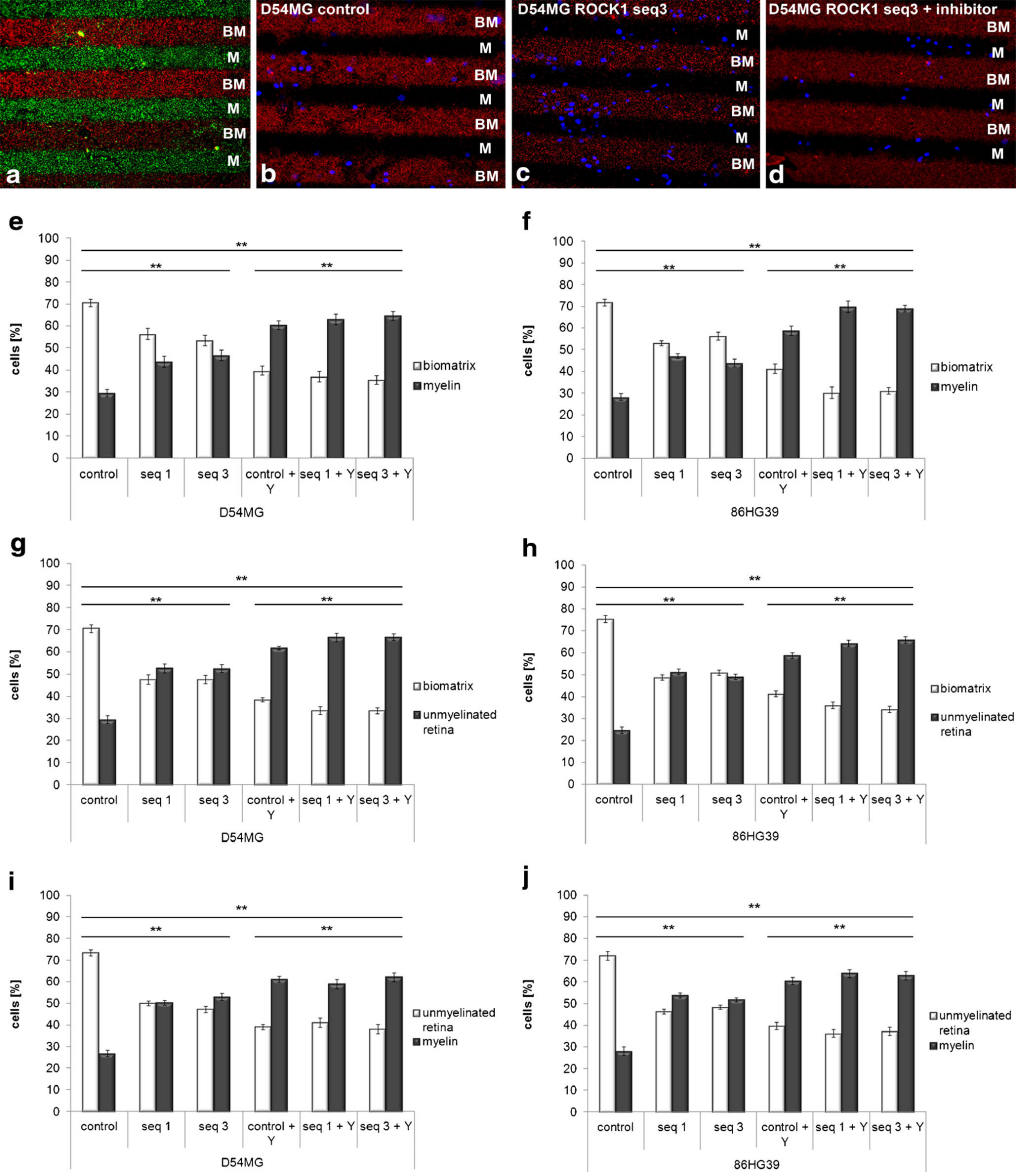
Quantification of D54MG and 86HG39 cells with altered ROCK1 expression and effects of the addition of the ROCK inhibitor Y27632 on preferences toward different substrates in the stripe assay. **a** Immunohistological staining revealing the structure of two alternate stripes, biomatrix (*BM*, *red*) vs. myelin (*M*, *green*), without cells. **b** D54MG cells (nuclei stained in *blue*) displaying a 70:30 distribution on BM (*red*) vs. M (no staining). **c** ROCK1 knockdown D54MG cells switched the substrate preference to a 55:45 distribution. **d** Additional administration of Y27632 led to a 35:65 distribution. Changes in cell distribution for ROCK1 knockdown D54MG cells plus ROCK inhibitor growing on BM vs. myelin (**e**), BM vs. unmyelinated retina (**g**), and myelin vs. unmyelinated retina (**i**). The same setting was used with ROCK1 knockdown 86HG39 glioma cells with ROCK inhibitor growing on BM vs. myelin (**f**), BM vs. unmyelinated retina (**h**), and myelin vs. unmyelinated retina (**j**) (*p < 0.05; **p < 0.001, *n* = 3, mean ± SEM)

The untreated control cells of both cell lines used (D54MG and 86HG39) again exhibited a distinct preference for BM, followed by myelin. All of the ROCK1 knockdown clones of these cell lines with two different shRNA vector sequences changed their preference for BM when tested against myelin. BM and myelin contained 70.4 ± 1.6 and 29.6 ± 1.63 % of the D54MG control cells, respectively. The ROCK1 knockdown clone seq1 seemed to lose preference for BM since the cells were distributed similarly on the two types of stripe (56.3 ± 2.51 % on BM vs. 43.7 ± 2.51 % on myelin). Comparable results were obtained for clone seq3, with 53.3 ± 2.44 % of the cells being located on BM and 46.7 ± 2.44 % on myelin. The application of Y27632 to ROCK1 knockdown cells resulted in a complete reversal of their preference. Even in the Y27632-treated control cells (i.e., without ROCK1 knockdown), only 39.7 ± 1.93 % of the cells were located on BM while 60.3 ± 1.93 % were on myelin. This change in substrate preference was even more marked in the Y27632-treated ROCK1 knockdown cells (seq1, 36.9 ± 2.44 % on BM vs. 63.1 ± 2.44 % on myelin; seq3, 35.5 ± 2.02 % on BM vs. 64.5 ± 2.02 % on myelin). This indicates that while ROCK1 alone can influence the substrate specificity, the inhibition of both ROCKs not only changes the specificity from approximately 70/30 to 50/50 but also completely switches the cells’ preference toward myelin. The 86HG39 cell line yielded comparable results (Fig. 5), with a switch of the cells’ preference from BM to myelin with the ROCK inhibitor and ROCK1 knockdown clones compared to untreated 86HG39 control cells.

Comparison of BM and the retina revealed a preference of untreated D54MG glioma cells for BM (70.6 ± 1.78 % for BM vs. 29.5 ± 1.78 % for retina). Changing the expression of ROCK1 resulted in an alteration of the substrate preference of the ROCK1 knockdown cells toward the retina. Both ROCK1 knockdown clones exhibited a cell distribution of 47.5 ± 2.1 % in BM vs. 52.5 ± 2.1 % in the retina for clone seq1 and of 47.5 ± 1.8 % on BM vs. 52.5 ± 1.8 % on the retina for clone seq3. The switching of the substrate preference was more pronounced following the addition of Y27632 to the ROCK1 knockdown cell clones, whereby the cell distributions were 33.5 ± 1.9 % on BM vs. 66.5 ± 1.9 % on the retina for clone seq1 and 33.4 ± 1.5 % on BM vs. 66.6 ± 1.5 % on the retina for clone seq3. Again, the 86HG39 glioblastoma cell line behaved in a similar way, such that 73.3 ± 1.6 % of the untreated cells were found in BM and only 24.7 ± 1.6 % of the cells were located in the retina. Knockdown of ROCK1 resulted in a change in the preferred cell location toward the retina: 48.7 ± 1.3 % on BM vs. 51.3 ± 1.3 % in the retina for clone seq1 and 51.0 ± 1.2 % in BM vs. 49.1 ± 1.2 % in the retina for clone seq3. The inhibitor Y27632 enhanced the effect of ROCK1 knockdown alone to distributions of 35.9 ± 1.5 % in BM vs. 64.1 ± 1.5 % in the retina for clone seq1 and of 34.1 ± 1.5 % in BM vs. 65.9 ± 1.5 % in the retina for clone seq3.

To clarify the preference of the cells toward myelin, in a third step, the retina was compared with purified myelin. In this approach, ROCK1 inhibition also led to a switch of the substrate specificity of all tested ROCK1 clones in both cell lines used (Fig. 5i, j). An additional dose of 100 μM Y27632 led to a further increase in the change in substrate specificity of the ROCK1 knockdown clones (Fig. 5i, j). Whether these additive effects of the ROCK inhibitor Y27632 are attributable to a combination of ROCK1 and ROCK2 inhibition or are purely the effect of a nearly 100 % inhibition of ROCK1 warrants further investigation.

**Fig. 6:**
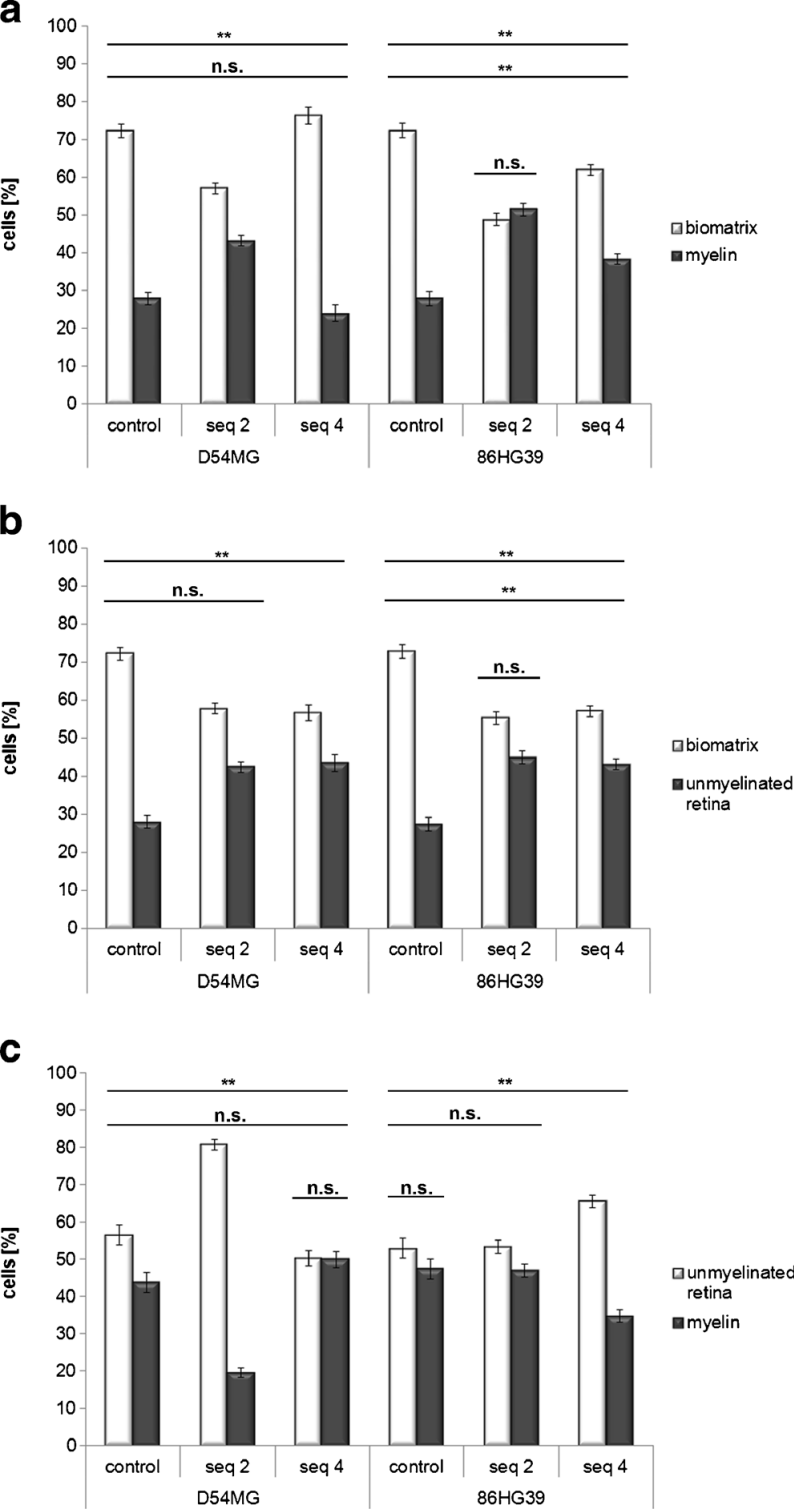
Influence of ROCK2 knockdown on cell substrate preferences using the stripe assay. **a** Testing Biomatrix vs. myelin using ROCK2 knockdown D54MG and 86HG39 cells revealed only slight changes in cell preference with altered ROCK2 expression. **b** For biomatrix vs. unmyelinated retina, ROCK2 knockdown led again to a slight switch of about 10 % in cell preference. **c** Testing myelin vs. unmyelinated retina revealed a substrate preference change in only one sequence of each cell line from a 55:45 to a 75:25 distribution toward myelin (*p < 0.05; **p < 0.001, *n* = 3, mean ± SEM)

The cell lines with a stable ROCK2 knockdown exhibited only slight changes in substrate preference compared to the control cells. Comparison of the BM with myelin substrates for the ROCK2 knockdown D54MG cells revealed cell contributions (BM vs. myelin) of 72.3 ± 1.74 % vs. 27.7 ± 1.74 % for control cells, 57.0 ± 1.41 % vs. 43.0 ± 1.41 % for seq2, and 76.2 ± 2.15 % vs. 23.8 ± 2.15 % for seq4. The 86HG39 cell line displayed a similar distribution of cells on the two substrates (BM vs. myelin): 72.3 ± 1.86 % vs. 27.7 ± 1.86 % for control cells, 48.7 ± 1.70 % vs. 51.3 ± 1.70 % for seq2, and 61.8 ± 1.44 % vs. 38.2 ± 1.44 % for seq4. In contrast to ROCK1 knockdown, only slight or no changes in cell distribution were observed in ROCK2 knockdown cells (Fig. 6a).

Testing BM against the retina revealed a preference for BM in control cells (BM vs. retina: 72.1 ± 1.74 % vs. 27.9 ± 1.74 % and 72.7 ± 1.86 % vs. 27.3 ± 1.86 %) and a slight shift but no significant change in preference for all four ROCK2 knockdown clones, to 57.10 ± 0.53 % vs. 42.90 ± 0.53 % (BM vs. retina). Furthermore, ROCK2 knockdown enhanced the preference toward the retina, but did not induce the same change in substrate preference as found for ROCK1 knockdown (Fig. 6b). The cell distributions were 56.4 ± 2.67 % vs. 43.6 ± 2.67 % (D54MG control), 80.6 ± 1.33 % vs. 19.4 ± 1.33 % (D54MG seq2), 50.1 ± 2.15 % vs. 49.9 ± 2.15 % (D54MG seq4), 52.8 ± 2.62 % vs. 47.2 ± 2.62 % (86HG39 control), 53.2 ± 1.87 % vs. 47.8 ± 1.87 % (86HG39 seq2), and 65.4 ± 1.60 % vs. 34.6 ± 1.60 % (86HG39 seq4), indicating no changes in substrate specificity in ROCK2 knockdown cells.

### Signaling of ROCK Knockdown

**Fig. 7:**
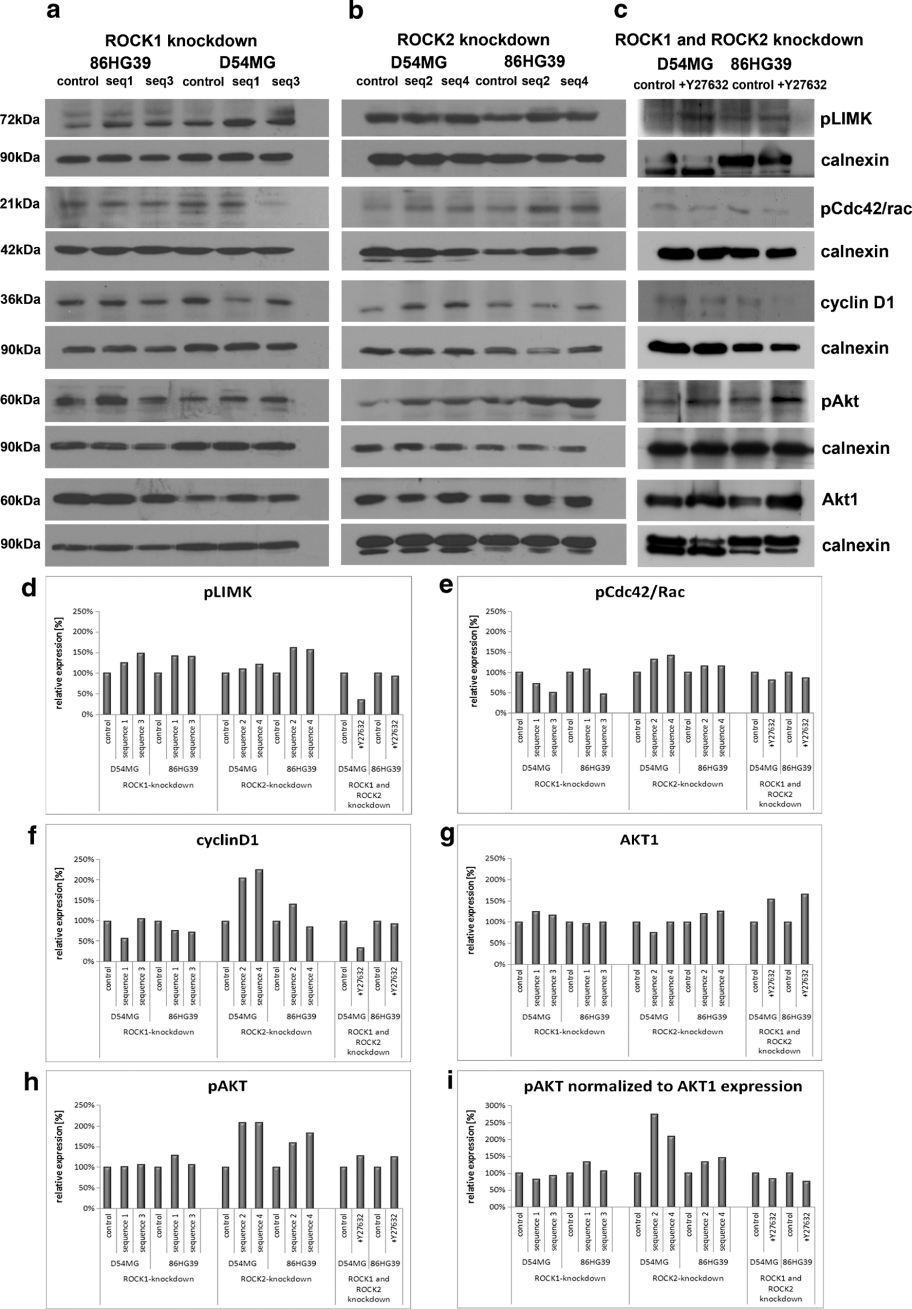
Signaling pathway analyses of cells with altered ROCK1 and ROCK2 expressions. The expression levels of several different proteins in the ROCK1 knockdown cells (**a**), ROCK2 knockdown cells (**b**), and cells treated with Rho kinase inhibitor Y27632 (**c**) are shown. Densitometric measurement of protein expression of phosphoLIMK (**d**), phosphoCDC42/Rac (**e**), cyclinD1 (**f**), AKT1 (**g**), and phosphoAKT (**h**) displayed different influences of ROCK1 and ROCK2 on protein expression. Using Y27632, we found nearly the same protein regulation as in cells with only ROCK1 knockdown

**Fig. 8:**
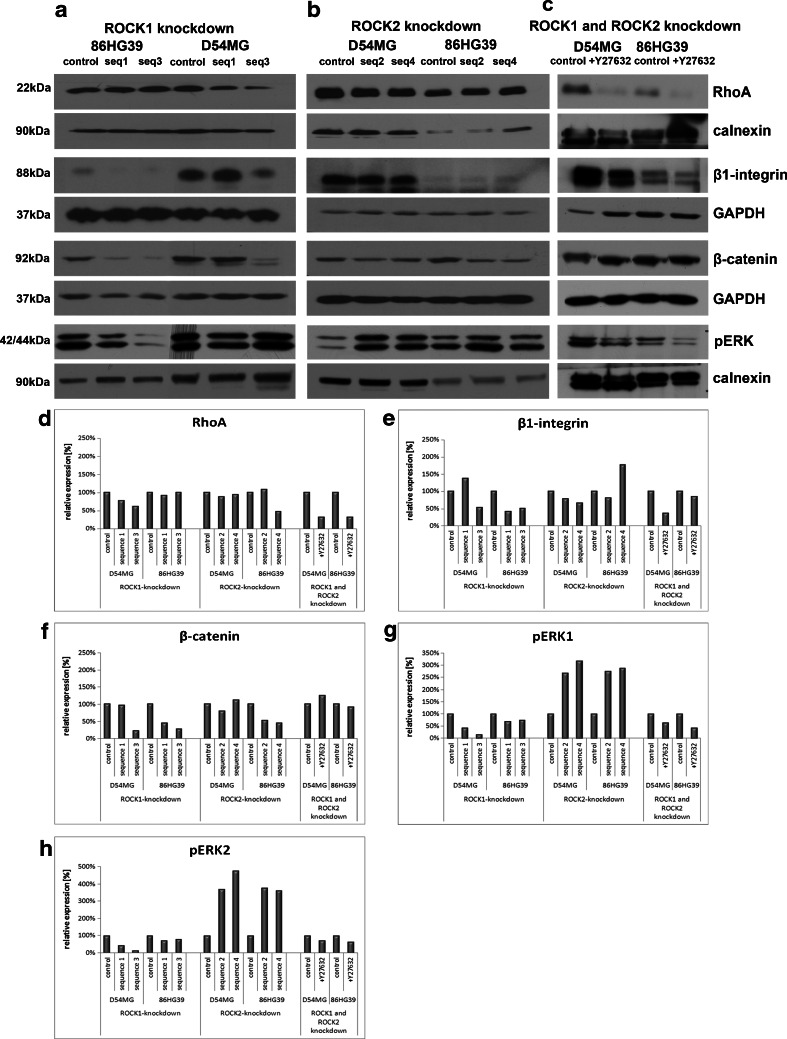
Second part of signaling pathway analyses of cells with altered ROCK1 and ROCK2 expressions. Here, we analyzed the protein expression of RhoA (**d**), β1-integrin (**e**), β-catenin (f), pERK1 (**g**), and pERK2 (**h**) using densitometric measurement in cells with ROCK1 knockdown (**a**), ROCk2 knockdown (**b**), and cells treated with Rho kinase inhibitor Y27632 (**c**)

The pathways involved in the effects of ROCK knockdown in glioblastoma cells were analyzed using Western blot analysis to determine the protein expression levels of phosphoLimK, phosphoRac1/cdc42, cyclin D1, Akt1, phosphoAkt, β1-integrin, β-catenin, phosphoERK1/2, and RhoA in cells with a stable ROCK1 and ROCK2 knockdown (Fig. 7a, b) as well as in glioma cells treated with 100 μM of Rho kinase inhibitor (Fig. 7c). Although a reduced level of ROCK2 was observed in cells with a stable ROCK1 knockdown, indicating a relationship between the expression levels of ROCK1 and ROCK2, downregulation of ROCK2 did not affect the expression level of ROCK1 (Fig. 2c, d). ROCK1 and ROCK2 affected the regulation of phosphoRac1/cdc42 (ser71) differently, with a reduction of ROCK1 leading to a decrease in the phosphorylation status of Rac1/cdc42 in three out of four clones, whereas a reduction of ROCK2 had the opposite effect in all four clones. Only 86HG39 seq1 cells yielded an enhancement in the phosphorylation of Rac1/cdc42 under ROCK1 knockdown. ROCK1 knockdown reduced cyclin D1 expression, except line 86HG39 seq1, and ROCK2 knockdown increased cyclin D1, except 86HG39 seq4. Using pERK1/2, we could show that ROCK1 knockdown leads to an inhibition of ERK activity and that ROCK2 knockdown leads to the opposite effect and enhances the phosphorylation of ERK1/2 in all tested clones (Fig. 8g, h).

ROCK1 knockdown slightly affects Akt1 expression, while phosphorylated Akt1 normalized to Akt1 expression displayed a reduction in ROCK1 knockdown clones D54MG seq1 and seq3, but an increase in all four ROCK2 knockdown clones (Fig. 7i). Using the inhibitor Y27632, both cell lines—D54MG and 86HG39—show a reduction in phosphoAkt1 expression compared to the control cells.

ROCK1 and ROCK2 knockdown decreased the expression of RhoA in the D54MG cell line; the 86HG39 cell line showed inconsistent results. Both ROCK1 and ROCK2 displayed an influence on β1-integrin and β-catenin expressions. The reduction of ROCK1 or ROCK2 expression leads in three out of four tested clones to a reduction in β1-integrin and β-catenin expressions as well. By inhibiting both kinases, β1-integrin protein expression was reduced in D54MG and 86HG39 cell lines, but β-catenin expression shows a reduction of expression only in the cell line 86HG39, not for the cell line D54MG (Fig. 7 d–f).

Using the glioma cell lines D54MG and 86HG39 treated with 100 μM Rho kinase inhibitor, nearly all tested proteins displayed the same regulation as in the cells with ROCK1 knockdown. Only for β-catenin did we find no significant changes in expression in the cell line D54MG treated with Y27632.

## Discussion

This study has shown that inhibition of ROCK1 and ROCK2 has a significant and differential influence on cell migration and proliferation. ShRNA was used to downregulate ROCK1 and ROCK2 expressions in the D54MG and 86HG39 glioblastoma cell lines, and the effects on cell migration, proliferation, substrate-dependent migration, and signaling pathways were compared. After verifying the knockdown at both the mRNA and protein levels in all the used cell lines and clones for both ROCK1 and ROCK2 knockdown, we examined changes in cell shape and morphology. There was no phenotypic difference between ROCK1 and ROCK2 knockdown cells with respect to morphology, indicating that the two ROCKs exert comparable influences on the cell cytoskeleton.

Inhibition of ROCK1 resulted in a decreased proliferation, whereas inhibition of ROCK2 had the opposite effect, significantly enhancing proliferation relative to the control cells and regulating cyclin D1, whose role is also apparent in fibroblasts [22], corneal epithelial cells, and hepatic stellate cells [23, 24], to mediate the canonical Wnt/TCF pathways involving β-catenin [25, 26]. In contrast to the opposing effects of the ROCKs described here, only ROCK2 was involved in cell proliferation changes in SH-SY5Y cells [27], indicating different pathways among cell lines. The influence of both Rho kinases on proliferation was additionally shown by analyzing the expression of EKR1/2 phosphorylation. ROCK1 knockdown reduces the level of phosphorylation, whereas ROCK2 knockdown leads to an increase in ERK activity.

Expanding on the effects on cell proliferation, we analyzed the effects of ROCK1 and ROCK2 knockdown on cell migration using different migration substrates that are natural partners of migrating cells within the brain. Strikingly, a significant increase in cell migration for both ROCK1 and ROCK2 knockdown cells using the wound-healing scratch assay was shown, contrasting previous reports using different inhibitors [28, 29]. ROCK1 knockdown cells plus the ROCK inhibitor Y27632 migrated more slowly than the control cells on laminin, whereas cells with a ROCK2 knockdown migrated faster than the control and ROCK1 knockdown cells. These findings indicate that the migration effect of ROCK1 is substrate-dependent, while that of ROCK2 is not.

This substrate dependency of glioma cells with altered ROCK expression was further scrutinized by conducting experiments with a stripe assay [21, 30, 31]. Untreated cells exhibited a significant substrate preference for BM [17]. ROCK1 knockdown cells changed their preference and migrated preferably toward unmyelinated retina and myelin compared to BM. This ROCK knockdown-induced preference toward myelin is not surprising since a hallmark of glioma migration is the long-distance movement of these cells along myelin-rich white matter tracts [3, 5, 32]. This behavior was confirmed in the stripe assay showing that reduced ROCK1 also changed the substrate preference of the cells. Addition of the ROCK inhibitor Y27632 to ROCK knockdown cells led to an almost complete switch in substrate preference toward the myelinated substrate, indicating an additive pharmacological effect, while the results of the same setup with ROCK2 knockdown cells were not as distinct. Indeed, comparison of BM and myelin as migration substrates revealed that only one shRNA sequence out of several tested in both cell lines changed the specificity from a 70/30 to a 55/45 distribution of cells. This finding indicates that the substrate dependence of glioma cells is mediated by ROCK1, not by ROCK2. Alternatively, a balanced and simultaneous regulation of both kinases may be in play, given that ROCK1 knockdown also influences ROCK2 expression, and not conversely, and cells with ROCK1 knockdown displayed the same regulatory effects on migration and proliferation as those treated with Y27632. Furthermore, analyzing downstream pathways in cells treated with Rho kinase inhibitor Y27632, we found in nearly all examined proteins the same regulation as in cells with ROCK1 knockdown. Although the two ROCK isoforms display an 80 % homology, ROCK1 is likely operating upstream of ROCK2 and is the key regulator of the activity of both kinases.

A driving force of cell movement is essential for cell migration, and this force is provided mostly by reorganization of the cytoskeleton, with directed protrusions at the front of the cells and cell detachment at the rear [9, 33, 34]. This reorganization of the cytoskeleton is mediated by members of the Rho family of GTPases, such as Rho, Rac, and Cdc42 [35]. Comparison of the expression of Cdc42/Rac in cells with ROCK1 and ROCK2 knockdown revealed that a reduction of ROCK1 also reduces the phosphorylation of Cdc42/Rac, but a reduction of ROCK2 increases the phosphorylation of Cdc42/Rac. Rac and Cdc42 regulate the polymerization of actin through the activation of Scar/WAVE and WASP/N-WASP complexes [36, 37], while phosphorylation of cdc42/rac1on Ser71 is assumed to attenuate the actin-driven motility. A decreased phosphorylation was observed in ROCK1 knockdown clones in the present study, together with reduced migration and proliferation, indicating the drastic remodeling of the cell morphology rather than changes in migration capability. As anticipated, inhibition of the expression of ROCK2 led to the opposite effect on Cdc42/Rac, with increased phosphorylation of Cdc42/Rac associated to increased migration.

Interestingly, both ROCK1 and ROCK2 knockdown resulted in a decreased RhoA protein. This suggests the presence of a feedback loop of ROCK expression on RhoA since ROCK1 and ROCK2 are downstream effectors of RhoA in glioma cells. This pathway may be regulated by phosphorylation, and thereby inactivation of p190 Rho GTPase-activating protein (p190A RhoGAP), since it was shown that in smooth muscle cells, ROCKs are involved in the induction of RhoA activity through the phosphorylation of p190A RhoGAP [38].

Dynamic regulation of the actin cytoskeleton is the main factor in cell motility and cell division, involving the phosphorylation of LIMK. The activation of ROCK by Rho leads to the LIMK-mediated inactivation of cofilin and results in the accumulation of actin and the formation of lamellipodia by inhibiting the actin depolymerization function of cofilin [39]. Interestingly, inhibition of ROCK1 and ROCK2 in the present study led to enhanced phosphorylation of LIMK in both cell lines and in all of the tested shRNA sequences. Rac also activates LIMK, thereby affecting the phosphorylation of cofilin, but reduces actomyosin-based cell contractility [40, 41]. The enhancement of LIMK phosphorylation in the ROCK1 knockdown cells might therefore occur by enhanced activation of Rac. Since ROCK2 knockdown leads to the inactivation of Cdc42/Rac, the enhanced LIMK phosphorylation might be based on a different pathway and may be the result of reduced ROCK1 expression, which is unaffected in ROCK2 knockdown cells.

The upstream factors involved in the effects of ROCK knockdown were identified by further analyzing the expression of β1-integrin. Integrin clustering and focal complex formation require the activity of Rho family members [42] such as p190A RhoGAP, which disrupts integrin clustering [43]. Interestingly, the influence of integrins on various cell activities such as actin remodeling and proliferation is not limited to one direction. The integrin ligand-binding activity can also be regulated from signals inside the cells [44, 45]. Cell signaling from inside-out to integrins is thought to be mediated by phosphatidylinositol 3-kinase [46] regulated by ROCK [47–49]. This regulation is confirmed in the present study which shows that ROCK1 knockdown leads to a decrease in β1-integrin expression and a slight increase in Akt expression, without significantly affecting Akt phosphorylation. We observed similar changes in β1-integrin expression in ROCK knockdown cells, but also a significant progression of Akt phosphorylation mediated by ROCK2, but not by ROCK1.
